# Acute and Subacute Safety Evaluation of Black Tea Extract (Herbt Tea Essences) in Mice

**DOI:** 10.3390/toxics10060286

**Published:** 2022-05-27

**Authors:** Xiaoyan Ding, Changshun Han, Weiping Hu, Chengqing Fu, Yixi Zhou, Zheng Wang, Qingyan Xu, Rongfu Lv, Chengyong He, Zhenghong Zuo, Jiyi Huang

**Affiliations:** 1 State Key Laboratory of Cellular Stress Biology, School of Life Sciences, The Fifth Hospital of Xiamen, Xiang’an Branch of the First Affiliated Hospital, Xiamen University, Xiamen 361102, China; dingxiaoyan199807@126.com (X.D.); changshunhan1999@126.com (C.H.); fcq0622@126.com (C.F.); yixizhou92@163.com (Y.Z.); wangzheng10202@126.com (Z.W.); xuqingyan@xmu.edu.cn (Q.X.); hecy@xmu.edu.cn (C.H.); 2 First Affiliated Hospital of Xiamen University, Xiamen 361003, China; hwp1227@126.com; 3 Xiamen Herbt Biotechnology Company Limited, Xiamen 361005, China; rongfu@Kaisonxm.com

**Keywords:** safety assessment, tea extract, theabrownin

## Abstract

Theabrownin (TB) is a heterogeneous biomacromolecule, extracted from tea, with many functional groups. Importantly, TB possesses diverse health benefits, such as antitumor activity and blood lipid-lowering effects. Presently, the content of TB in tea extract is relatively low. Here, we obtained a deep-processed black tea extract with a high content of TB (close to 80%), which was named Herbt Tea Essences (HTE). Currently, this study was designed to evaluate the biosafety of high-content TB products on mice. We implemented acute and subacute toxic experiments to assess its safety on organs, the serum biochemical and molecular levels. In the acute exposure study, we found that the median lethal dose (LD_50_) value of HTE was 21.68 g/kg (21.06–24.70 g/kg, greater than 5 g/kg), suggesting that HTE had a low acute toxicity. In the 28-day subacute exposure study, our results showed that no abnormal effects were observed in the 40 and 400 mg/kg/day HTE-treated groups. However, we observed slight nephrotoxicity in the 4000 mg/kg/day HTE-treated group. The HTE-induced nephrotoxic effect might involve the inflammatory response activation mediated by the nuclear transcription factor kappa-B (NF-κB) signaling pathway. This study would provide valuable data for the TB safety assessment and promote this natural biomacromolecule application in daily drinking.

## 1. Introduction

Tea is one of the most widely consumed beverages around the world. Two-thirds of the global population has the habit of regularly drinking tea [1]. Black tea is a fully fermented tea and is rich in polyphenols [2,3]. Through fermentation, catechins, the main active derivative of polyphenols, are oxidized into complex tea pigments. Tea pigments are composed of theaflavin (TF), thearubigin (TR) and theabrownin (TB). Among them, TF and TR are further oxidized, polymerized and condensed to form TB [4,5]. It is a heterogeneous macromolecule with many benzene rings, hydroxyl functional groups, carboxyl groups, methyl groups and amino groups [6], which has many health benefits, such as antitumor activity and blood lipid-lowering effects [7,8]. Therefore, it is the major mediator of the pharmacological effects of fermented tea [9]. Currently, the detailed content of TB in tea extract is unclear [10]. Thus, people are encouraged to improve the ratio of TB in fermented tea [11].

In this study, we deeply processed black tea and obtained a product with a high content of TB and small portions of tea polyphenols (TPs) and caffeine, which was named Herbt Tea Essences (HTE). HTE was made by water extraction and fermentation and contained a high concentration of TB. Due to the high proportion of beneficial ingredients, it is expected to have benefits from both health and market perspectives.

Due to the differences of the fermentation processes, the main ingredients of tea are different, so its biological impacts on human health are also different [12]. When mice were exposed to a high dose of tea extract, mild hepatotoxicity and nephrotoxicity can be observed [13]. A previous clinical research report showed that the excessive consumption of black tea is harmful to human beings [14]. Thus, the safety of tea and processed tea products should be given more attention.

To ensure safe consumption, it is necessary to carefully assess its safety and to estimate the safe threshold of intake to ensure the health safety of the population and to promote its use. Therefore, we first analyzed the composition of HTE. Then, we conducted acute and subacute oral administration studies of HTE in mice and measured their mortality, the median lethal dose (LD_50_) value, organ somatic index and serum biochemical parameters.

## 2. Materials and Methods

### 2.1. Chemicals

HTE was obtained from Xiamen Herbt Biotechnology (Xiamen, China). Food maltodextrin (CAS 9050-36-6) was purchased from Jinan Luhui Chemical Co., Ltd. (Jinan, China). All other analytical grade chemicals in this study were obtained from commercial sources.

### 2.2. Preparation of HTE

Ceylon black tea (Sri Lanka) was soaked in pure water for several hours, filtered and removed slag to obtain black tea extraction water. The leaching water was separated by membrane separation technology to remove most of the unwanted components. Then, we employed a C18 (100 mm × 4.6 mm, 5-μm particle size) column filtration to separate and purify the liquid. Repeating the process of membrane separation and column filtration until the contents of TB, TPs and caffeine reached the standard spray-dried (Lemar, Changzhou, China), we obtained a pure dry powder and 1:1 blended dextrin as the auxiliary material. Through the above steps, we obtained the HTE products.

### 2.3. Content Determination of Components in HTE

#### 2.3.1. Determination of Caffeine Content

According to the method of China standard (QB/T 4067–2010), a proper amount of HTE was dissolved in distilled water, and hydrochloric acid and basic lead acetate solution was added. After filtration, a sulfuric acid solution was added and diluted with distilled water. After filtration, the filtrate was taken to measure the absorbance (A) at 274 nm using a spectrophotometer (Shanghai Spectrum Co., Ltd., Shanghai, China), and distilled water was used as a blank control. The calculation was performed according to the following formula [15]:

the content of caffeine (HTE g/100 g) = c × 2 ÷ m

where c and m represent the concentration and mass, respectively.

#### 2.3.2. Determination of TB Content

The sample was weighed and dissolved in distilled water. N-butanol, oxalic acid solution and 95% ethanol were added. Next, the absorbance value (A) was measured at a wavelength of 279 nm using a spectrophotometer (Shanghai Spectrum Co., Ltd., Shanghai, China), and 95% ethanol was used as the blank solution. Three parallel experiments were conducted simultaneously, and the calculations used the following formula [16]:

the content of TB (HTE g/100 g) = (A + 0.0038) × 106/(9.4893 × m × 103)

where m represents the mass, and A represents absorbance.

#### 2.3.3. Determination of the TP Contents

The contents of the TPs were determined according to the method of the China standard (QB/T 4067-2010). An appropriate amount of sample was dissolved in distilled water. After ferrous tartrate solution and phosphate buffer solution (PBS, pH = 7.5) were added, the solution to be tested was obtained. The absorbance of the tested solution (A1) and 25 mL of the tested solution supplemented with 4 mL of distilled water (A2) was measured at a wavelength of 540 nm using a spectrophotometer (Shanghai Spectrum Co., Ltd., Shanghai, China). Phosphate buffer was used as a control. The contents of the TPs (HTE g/100 g) = (A1 − A2) × 1.975 × 200/(30 × m) [15], and m represents mass. A1 andA2 represents absorbance.

### 2.4. Mice

Kunming mice (male:female = 1:1, 20–25 g) were obtained from Beijing Vital River Laboratory Animal Technology Co., Ltd. (Beijing, China). All mice were specific pathogen-free grade (SPF) and were maintained in a temperature of 22 ± 1 °C with a 12-h light/dark cycle and a relative humidity of 55 ± 5% and allowed to access a standard diet and water (standard rodent chow diet, Keao Xieli Feed Company, Beijing, China). Mice underwent acclimatization feeding for 3 days before experiments. All animal experiments were conducted and approved by the Xiamen University Institutional Animal Ethics Committee (Acceptance No. XMULAC20170361).

### 2.5. Experiment of Acute Administration

Based on the GB15193.3-2014 national standard method, an acute oral administration test was carried out. Five dose groups (12, 14, 17.7, 20.2 and 24 g/kg) were set up for HTE (*n* = 10). Two intragastric gavages with an interval of 4 h were performed. After oral administration, the manifestations of the animals within 14 days were continuously observed, including their signs of poisoning (the degree and duration) and the number and timing of animal deaths, and then, the median lethal dose (LD_50_) was calculated with Karber’s method. We obtained 17 and 20 g/kg mouse serum and tissues (*n* = 6). The kidney and liver somatic indexes were calculated and serum biochemical indices were tested. We used the equation: lgLD_50_ = ∑ [(Xi + Xi + 1) × (Pi + 1 − Pi)/2] to calculate LD_50_. Xi and Xi + 1 are the logarithms of dose in two adjacent groups. Pi + 1 and Pi are the mortalities in two adjacent groups.

### 2.6. Experiment of Subacute Administration

According to the GB15193.3-2014 national standard method of China, we conducted a 28-day gavage administration test. The specification of the HTE products was 2 g per pack (1 g dextrin and 1 g black tea extract). The adults were given one pack per day, 33.3 mg/kg/day. The dose conversion factor from humans to mice was 12.33 [17,18]. Therefore, we concluded that the gavage dose for mice was 400 mg/kg/day. Two doses of 40 and 4000 mg/kg/day were set in accordance with a gradient of 10 times. Sterile water was used as a comparison. Each group consisted of 20 mice, half male and half female. After treatment for 28 days, all mice were sacrificed and sampled. Blood was collected to detect their blood biochemical indicators, and their organs were collected to detect any toxic effects and to conduct subsequent experiments.

### 2.7. Serological Assays

Serum was separated from the whole blood using a centrifuge for 15 min at 3000× *g*. Then, the serum biochemical indices were measured with a Cobas 8000 c702 module analyzer (Roche, Rotkreuz, Switzerland), including the levels of aspartate aminotransferase (AST), alanine aminotransferase (ALT), uric acid, blood glucose (GLU), blood urea nitrogen (BUN), creatinine (CREA), alkaline phosphatase (ALP), triglycerides (TG), albumin (ALB), globulin (GLO), total protein (TP) and very low-density lipoprotein (VLDL).

### 2.8. Kidney Staining

Kidney samples were fixed in 4% paraformaldehyde dissolved in PBS and encased in paraffin. The paraffin blocks were sliced into 5-μm-thick sections. We used hematoxylin and eosin (HE) to stain the sections and imaged them with a microscope (Leica, DM4B; Wentzler, Germany). According to our previous method [19], we calculated the number of glomeruli and the relative area of the glomeruli and renal tubules by using Image-Pro Plus 6.0 (Olympus, Tokyo, Japan).

### 2.9. Western Blot

The mice were sacrificed, and their kidneys were collected. We mixed the renal tissue with radioimmune precipitation assay (RIPA) buffer (Solarbio, Beijing, China), including a protease inhibitor cocktail (1:100) (MedChemExpress, Monmouth Junction, NJ, USA). Next, the above solution was centrifuged at 12,000× *g* for 15 min, and the supernatant was collected. The supernatant was mixed with sodium dodecyl sulfate (SDS) loading buffer with β-mercaptoethanol. Standard SDS-polyacrylamide gel electrophoresis (PAGE) was performed, and the proteins were transferred onto polyvinylidene difluoride (PVDF) membranes. The membrane was sealed in 5% milk dissolved in Tris-buffered saline solution with 0.05% Tween 20 (TBST). We analyzed the samples with the following primary antibodies: rabbit polyclonal anti-IKB-α, rabbit monoclonal anti-p-IKB-α, rabbit monoclonal anti-IKKα/β, rabbit polyclonal anti-p-IKKα/β (Beyotime, Shanghai, China), rabbit polyclonal anti-NF-κB p65 and rabbit polyclonal anti-p-NF-κB p65 (ZEN BIO, Chengdu China). The membranes were washed with TBST and then incubated with horseradish peroxidase (HRP)-conjugated secondary antibodies (BOSTER, Wuhan, China) (1:10000). The membranes were covered with a WesternBright electrochemiluminescence (ECL) substrate and imaged with a ChemiScope 5300 instrument (Clinx Science Instruments, Shanghai, China).

### 2.10. Real-Time Quantitative PCR (RT-qPCR)

Total RNA was extracted from mouse kidneys by using TRIzol (Accurate, Shanghai, China), and then, the RNA was reverse-transcribed into cDNA by the TransScript First Strand cDNA Synthesis SuperMix Kit (Accurate, Shanghai, China). We designed primers for the RT-PCR, as listed in Table S1. Then, we performed qPCR under the following conditions: 95 °C for 3 min, 40 cycles of 95 °C for 15 s, 60 °C for 15 s and 72 °C for 18 s by using TransStart Tip Green qPCR SuperMix (Transgen, Beijing, China). The melting curve was measured at 76–95 °C to evaluate the specificity of the primer. The levels of *Tnfa*, *Il1b* and *Il6* were quantified with the 2^−^^△△Ct^ method. All experiments were performed in triplicate, and mouse gapdh was utilized as a reference gene.

### 2.11. Statistical Analysis

All data were obtained from at least three independent experiments and statistically analyzed with the mean ± standard error of the mean (SEM). Unpaired *t*-tests were performed using GraphPad Prism 7.0 (GraphPad software, San Diego, CA, USA). A value of *p* < 0.05 was regarded as statistically significant. Organ somatic index = Organ weight/body weight × 100%.

## 3. Results

### 3.1. Identification of HTE Ingredients

Here, we want to obtain high-concentration TB tea products. According to the method in the national standard of China (QB/T 4067–2010), we detected the content of TB. We found that HTE contained 79% TB. What is more, TPs and caffeine were the most important active substances in water-extracted tea products [20]. Our results showed that HTE contained 8.46% TPs and 2.76% caffeine.

### 3.2. Acute Oral Administration Study

The experimental design and results are provided in Table 1. We carefully observed the toxic signs or symptoms in the mice after 4 and 12 h and then once a day for 14 days. Clinical observations of the mice prior to death noted hypoactivity and a lower body temperature. There were no adverse effects among the survivors during the 14 days after treatment with HTE. The LD_50_ value and its 95% confidence limit for HTE were 21.68 g/kg (21.06–24.70 g/kg) (Table 1). Its LD_50_ was much higher than 5 g/kg. According to GB 15193.3-2014, HTE was considered a nontoxic substance. Since the highest dose (24 g/kg) was lethal, we used 17.7 and 20.2 g/kg HTE as the exposure groups (minimum lethal doses) to further analyze the safety of HTE. Compared with the control group, the liver and kidney somatic index and serum biochemistry were not affected (Tables S2 and S3). These results indicated that 17.7 and 20.2 g/kg HTE exposure did not cause adverse effects on mice.

### 3.3. Subacute Oral Administration Study

#### 3.3.1. Effect of HTE on Body Weight Changes and Organ Somatic Index

**Figure 1:**
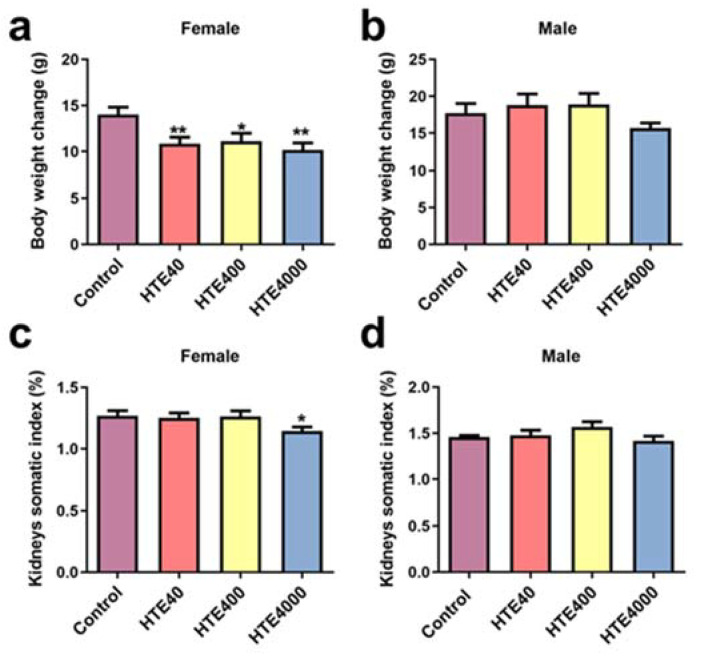
Effect of HTE (40, 400 and 4000 mg/kg) on the body weight and kidney somatic index in the subacute administration study. All data are presented as the mean ± SEM (*n* = 10). (**a**) The body weight change of females on the 28th day. (**b**) The body weight change of males after 28 days. (**c**) Kidney somatic index for females. (**d**) Kidney somatic index for males. Statistical significance was analyzed by using the Student’s *t*-test. Compared with the control, * and ** represent 0.01 < *p* < 0.05 and 0.001 < *p* < 0.01, respectively.

We found that, in the subacute oral administration experiment, the body weight change of female mice in the HTE40 (0.77-fold, *p* = 0.0086), HTE400 (0.79-fold, *p* = 0.0246) and HTE4000 (0.72-fold, *p* = 0.0028) groups were significantly lower than those of the control group (Figure 1a). However, there were no significant changes in the body weight of male mice (Figure 1b). Compared with the control group, the brain somatic index of female mice remarkably increased at the 40 mg/kg/day HTE-treated group (1.1-fold, *p* = 0.0473) (Table 2). Apart from this, after treatment with 40 and 400 mg/kg/day HTE, there were no statistically significant differences in the other organ somatic index of female and male mice compared with the control group (Table 2). The kidney somatic index of female mice decreased significantly at the 4000 mg/kg/day HTE-treated group (0.90-fold, *p* = 0.0294) (Figure 1c). The results showed that 4000 mg/kg/day of HTE exposure may have an adverse effect on the kidneys but did not adversely affect the other organs, such as the liver, heart, lungs and spleen.

#### 3.3.2. Effect of HTE on Serum Biochemical Parameters

**Figure 2:**
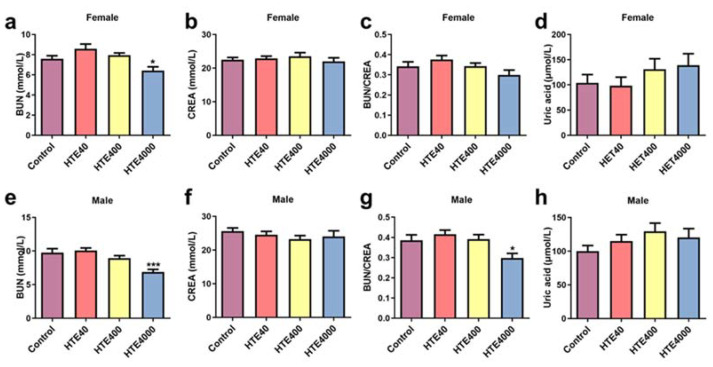
Effect of HTE (40, 400 and 4000 mg/kg) on the renal serum biochemical parameters in the subacute administration study. All data are presented as the mean ± SEM (*n* = 10). (**a**) The blood urea nitrogen (BUN) levels of female mice. (**b**) The creatinine (CREA) levels of female mice. (**c**) The BUN/CREA levels of female mice. (**d**) The uric acid levels of female mice. (**e**) The BUN levels of male mice. (**f**) The CREA levels of male mice. (**g**) The BUN/CREA levels of male mice. (**h**) The uric acid levels of male mice. Statistical significance was analyzed by using the Student’s *t*-test. Compared with the control, * and *** represent 0.01 < *p* < 0.05 and *p* < 0.001, respectively.

Compared with the control group, the BUN levels of female (0.84-fold, *p* = 0.0294) and male mice (0.7-fold, *p* = 0.0006) markedly declined at a dose of 4000 mg/kg/day (Figure 2a,e). The BUN/CREA levels of male mice decreased significantly (0.77-fold, *p* = 0.0222) (Figure 2g). With a dose of 4000 mg/kg/day HTE, the VLDL value among the males decreased significantly (0.77-fold, *p* = 0.001). The TG levels showed a significant decrease with HTE treatment doses of 400 (0.75-fold, *p* = 0.0499) and 4000 (1.37-fold, *p* = 0.0006) mg/kg/day in males (Table 3). In addition to this, compared with the control group, there was no statistically significant difference in the other serum biochemistry of female and male mice in the 40 and 400 mg/kg/day groups (Table 3). The results indicated that 4000 mg/kg/day of HTE exposure may have an adverse effect on renal function.

#### 3.3.3. The Histopathological Examination of the Kidneys

**Figure 3:**
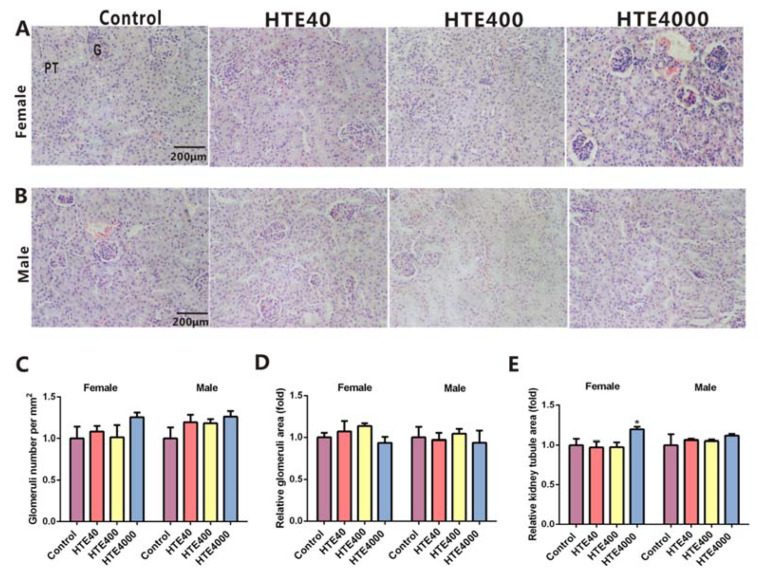
The histopathological examination of the kidneys in the HTE (40, 400 and 4000 mg/kg) subacute administration study. All data are presented as the mean ± SEM (*n* = 3). (**A**) Image of renal sections stained with HE for females. Scale bar = 200 μm. G and PT represents glomeruli and proximal tubule, respectively. (**B**) Image of renal sections stained with HE for males. (**C**) The number of glomeruli for females and males. (**D**) Relative area of kidney tubule for females and males. (**E**) Relative area of glomeruli for females and males. Statistical significance was analyzed by using the Student’s *t*-test. Compared with the control, * represents 0.01 < *p* < 0.05.

There was no difference in renal histomorphology for males and females among the control, 40, 400 and 4000 mg/kg/day groups (Figure 3A,B). Treatments with doses of 40 and 400 mg/kg/day HTE caused no significant changes in the number and area of the glomerulus or the relative kidney tubule area (Figure 3C–E). The relative kidney tubule area in females remarkably increased in the 4000 mg/kg/day HTE group (1.39-fold, *p* = 0.0265) (Figure 3D). The results further suggested that 4000 mg/kg/day of HTE exposure could cause mild damage to the kidneys.

#### 3.3.4. High Doses of HTE Exposure Promoted Inflammatory Response by Activating Renal NF-κB Signaling Pathway

**Figure 4:**
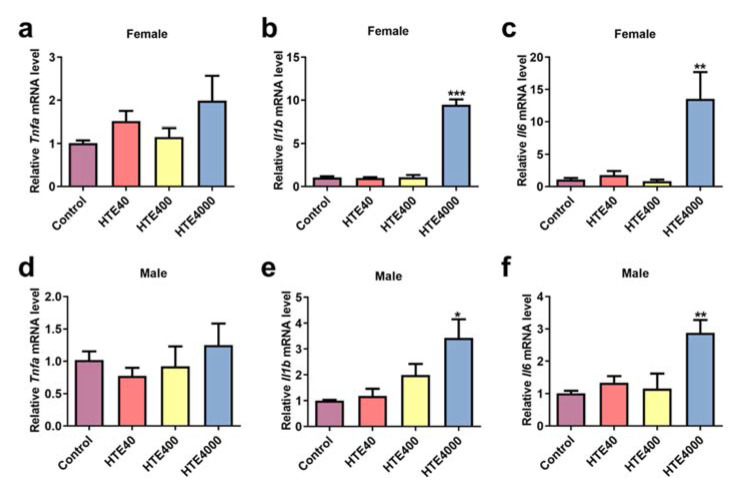
The relative mRNA expression of *Tnfa*, *Il1b* and *Il6* in the kidneys in the HTE (40, 400 and 4000 mg/kg) subacute administration study. All data are presented as the mean ± SEM (*n* = 6). The mRNA expression levels of (**a**) *Tnfa*, (**b**) *Il1b* and (**c**) *Il6* for females. The mRNA expression levels of (**d**) *Tnfa*, (**e**) *Il1b* and (**f**) *Il6* for males. Statistical significance was analyzed by using the Student’s *t*-test. Compared with the control, *, ** and *** represent 0.01 < *p* < 0.05, 0.001 < *p* < 0.01 and *p* < 0.001, respectively.

**Figure 5:**
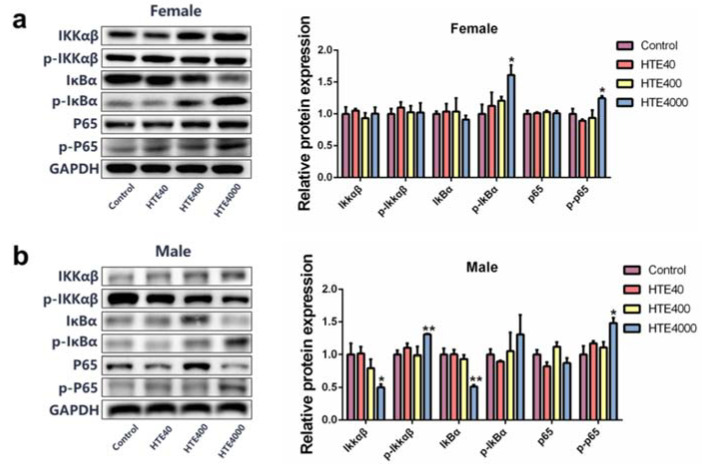
The relative protein expression of the NF-κB signaling pathway on the mice kidneys in the HTE (40, 400 and 4000 mg/kg) subacute administration study. All data are presented as the mean ± SEM (*n* = 3). (**a**) Expression of the NF-κB signaling pathway-related proteins in female mice. (**b**) Expression of the NF-κB signaling pathway-related proteins in male mice. Significance was analyzed by using the Student’s *t*-test. Compared with the control, * and ** represent 0.01 < *p* < 0.05 and 0.001 < *p* < 0.01, respectively.

As shown in Figure 4, at a dose of 4000 mg/kg/day HTE, the inflammatory cytokine *Il1b* of female (9-fold, *p* = 0.0001) and male (3.42-fold, *p* = 0.0283) mice and *Il6* of female (12.33-fold, *p* = 0.0026) and male (3.42-fold, *p* = 0.0283) mice levels in the kidney significantly increased. While the expression of *Tnfa* was not altered. Accordingly, we investigated the possible nephrotoxic mechanism. The critical role of the nuclear transcription factor kappa-B (NF-κB) family of proteins in inflammation is well-documented [21]. We detected the expression levels of NF-κB signaling pathway-related proteins in the kidneys. As shown in Figure 5, Compared with the control group, the relative protein expression of IKKα/β in the 4000 mg/kg/day HTE group of males decreased significantly (0.50-fold, *p* = 0.0475). Meanwhile, the phosphorylation of IKKα/β in males was significantly increased (1.31-fold, *p* = 0.007). The relative protein levels of IκBα in males significantly declined (0.51-fold, *p* = 0.007), and the relative protein levels of p-IκBα in females was significantly increased (1.61-fold, *p* = 0.0468). The relative protein expression of p-P65 in females (1.24-fold, *p* = 0.0507) and males (1.48-fold, *p* = 0.0336) was significantly increased (Figure 4a,b). These results suggested that 4000 mg/kg/day HTE exposure could cause an inflammatory response mediated by the NF-κB signaling pathway.

## 4. Discussion

Tea is one of the most popular drinks consumed around the world [22]. The main ingredient of fully fermented black tea is TB [23]. In this study, HTE is a fully fermented black tea product. Our results showed that HTE contained 79% TB. Tea with high TB is more beneficial to health [10]. Therefore, HTE is expected to have multiple potential health benefits and marketing prospects. Moreover, HTE is extracted by water extraction, and the remaining 9.78% may be water-soluble chemical components. After analyzing the compounds of HTE, to evaluate the safety of HTE, we conducted acute and subacute oral administration studies in mice.

The LD_50_ of tea polyphenols and caffeine in mice are 2640 and 127~248 mg/kg/day, respectively [24]. A previous study showed that the LD_50_ values of three typical Yunnan Pu’er teas are 9.7, 11.2 and 12.2 g/kg, respectively [25]. In our acute oral administration study, the LD_50_ value of HTE was 21.68 g/kg. Based on these studies, HTE was believed to be very safe for consumption.

In the subacute oral administration study, mice were intragastrically administered 40, 400 and 4000 mg/kg/day HTE for 28 days. In these three groups, we did not find any toxicity characteristics in the 40 and 400 mg/kg/day-treated groups. Interestingly, with the 400 mg/kg/day HTE exposure, the TG of male mice showed a reduction in the levels. In a previous study, TB and fermented tea were proved to reduce the levels of the total TG in mice [26]. In addition, another study also showed that black tea can ameliorate fructose-induced hyperlipidemia and hyperleptinemia [27]. Therefore, we deduced that HTE may also have an antihyperlipidemic effect that deserves to be studied more fully in the future.

When 4000 mg/kg/day HTE was administered, the kidney somatic index and renal function index exhibited slight damage to mice. This dose does not reflect any actual intake by the human body, so it is normal to observe a certain toxicity. Nonetheless, we explored its possible mechanism of causing mild nephrotoxicity. The inflammatory response plays a key role in kidney disease [28]. The NF-κB signaling pathway is closely related to the increase in inflammation levels [29]. NF-κB is inactive during normal states. When cells are stimulated by inflammatory factors, including IL-6 and IL-1β, NF-κB is activated, and P65 enters the nucleus [30,31]. P65 can bind to the nucleotide sequence in the promoter region of the inflammatory factor, and it is involved in the transcription of *Tnfa*, *Il6* and *Il1b*, thereby enhancing their expression [32,33].

In this study, 4000 mg/kg/day HTE exposure could induce an inflammatory response by activating the renal NF-κB signaling pathway. As a complex chemical mixture, which of its components contributed to the toxicity of 4000 mg/kg/day HTE? First of all, HTE is mixed with dextrin 1:1. According to the ratios of TPs and caffeine, 4000 mg/kg/day HTE contains approximately 169.2 mg/kg/day TPs and 55.2 mg/kg/day caffeine, and 50 mg/kg/day epigallocatechin gallate (EGCG), a typical TP, and 50-75 mg/kg/day caffeine can produce certain toxic side effects in mice [34,35]. It can be inferred that the renal toxicity caused by high doses in mice is mainly due to excessively high doses of TPs and caffeine.

When humans are considered as the consumer of foods or medicines, the reference dose (RfD) is an instructive parameter [36]. Based on safety considerations, the evaluation of RfD is generally first conducted in animals (mainly mice or rats) [37,38]. The results on animals are then extrapolated to humans [39]. We could conduct a 90-day oral toxicity study according to the Organization for Economic Cooperation and Development (OECD) Guideline 408 to provide better guidance for people who want to consume HTE daily [40].

## 5. Conclusions

In conclusion, our target was to identify a tea extract product with a high concentration of TB and to evaluate its biological safety. In the acute oral toxicity study, we obtained a LD_50_ value of 21.68 g/kg for HTE, indicating that HTE was practically nontoxic. In the subacute toxicity study, up to 400 mg/kg/day HTE was nontoxic. The nephrotoxic effect of HTE in the 4000 mg/kg/day group might activate the inflammatory response mediated by the NF-κB signaling pathway. This study provides effective knowledge for us to further promote the market-oriented upgrade of this tea product with natural biomacromolecular TB.

## Figures and Tables

**Table 1:** Acute toxicity dose design and mortality on mice. *n* represents the number of mice.

HTE Dose (g/kg)	Logarithm (Dose)	Sex	N	Mortality	Ratio
12	1.0792	♂	5	0	0%
		♀	5	0	
14	1.1544	♂	5	0	0%
		♀	5	0	
17.7	1.2297	♂	5	0	0%
		♀	5	0	
20.2	1.3049	♂	5	1	10%
		♀	5	0	
24	1.3802	♂	5	5	100%
		♀	5	5	

**Table 2:** Organ somatic index of HTE (40, 400 and 4000 mg/kg) at the end of the subacute toxicity test.

Organ Somatic Index (%)	Sex	Control	40 mg/kg	400 mg/kg	4000 mg/kg
Liver	♂	4.04 ± 0.07	3.84 ± 0.1	3.83 ± 0.07	3.86 ± 0.09
	♀	3.90 ± 0.18	3.97 ± 0.15	4.11 ± 0.19	4.01 ± 0.12
Brain	♂	1.15 ± 0.03	1.14 ± 0.03	1.13 ± 0.04	1.12 ± 0.01
	♀	1.32 ± 0.04	1.46 ± 0.02 *	1.40 ± 0.03	1.33 ± 0.03
Spleen	♂	0.21 ± 0.01	0.22 ± 0.01	0.21 ± 0.01	0.20 ± 0.008
	♀	0.30 ± 0.02	0.32 ± 0.03	0.31 ± 0.02	0.28 ± 0.02
Pancreas	♂	0.51 ± 0.07	0.47 ± 0.05	0.52 ± 0.09	0.57 ± 0.07
	♀	0.62 ± 0.05	0.66 ± 0.05	0.66 ± 0.03	0.64 ± 0.40
Lungs	♂	0.54 ± 0.008	0.54 ± 0.01	0.52 ± 0.01	0.54 ± 0.02
	♀	0.54 ± 0.01	0.55 ± 0.01	0.54 ± 0.01	0.54 ± 0.03
Heart	♂	0.62 ± 0.02	0.66 ± 0.02	0.62 ± 0.04	0.54 ± 0.02
	♀	0.50 ± 0.04	0.52 ± 0.05	0.57 ± 0.05	0.47 ± 0.03
Testes	♂	0.60 ± 0.006	0.56 ± 0.005	0.55 ± 0.008	0.60 ± 0.006
Epididymides	♂	0.18 ± 0.02	0.17 ± 0.02	0.18 ± 0.01	0.18 ± 0.03
Ovaries	♀	0.10 ± 0.01	0.08 ± 0.01	0.06 ± 0.01	0.09 ± 0.01
Uterus	♀	0.29 ± 0.02	0.33 ± 0.02	0.34 ± 0.03	0.27 ± 0.05

All data are presented as the mean ± SEM (*n* = 10). Statistical significance was analyzed by using the Student’s *t*-test. Compared with the control, * represents 0.01 < *p* < 0.05.

**Table 3:** The serum biochemical parameters, blood glucose and triglyceride of HTE (40, 400 and 4000 mg/kg) at the end of subacute toxicity study.

Parameter	Sex	Control	40 mg/kg	400 mg/kg	4000 mg/kg
ALT(U/L)	♂	39.1 ± 3.3	39.5 ± 1.8	38.8 ± 3.7	37.3 ± 3.6
	♀	35.0 ± 3.6	36.2 ± 2.1	33.3 ± 2.8	34.3 ± 1.6
AST(U/L)	♂	128.9 ± 6.1	127.0 ± 9.3	134.3 ± 11.4	124.8 ± 10.6
	♀	130.4 ± 9.6	139.1 ± 12.5	130.6 ± 13.9	134.0 ± 11.4
AST/ALT	♂	3.4 ± 0.2	2.8 ± 0.3	3.5 ± 0.2	4.0 ± 0.4
	♀	3.8 ± 0.3	3.3 ± 0.2	3.9 ± 0.1	3.5 ± 0.2
ALB (g/L)	♂	49.1 ± 0.7	47.0 ± 0.7	47.0 ± 0.7	49.8 ± 1.3
	♀	49.4 ± 0.7	51.8 ± 1.7	48.5 ± 0.7	48.2 ± 1.0
GLO (g/L)	♂	21.2 ± 0.3	20.6 ± 0.5	21.7 ± 0.4	19.8 ± 0.9
	♀	17.1 ± 1.0	16.3 ± 0.3	18.7 ± 1.1	16.2 ± 0.8
ALB/GLO	♂	2.3 ± 0.04	2.2 ± 0.06	2.1 ± 0.06	2.5 ± 0.09
	♀	2.94 ± 0.1	3.1 ± 0.09	2.6 ± 0.1	3.0 ± 0.1
TP (g/L)	♂	70.3 ± 0.89	67.7 ± 1.02	67.2 ± 0.86	69.7 ± 1.9
	♀	66.6 ± 1.5	68.2 ± 0.7	67.3 ± 1.4	64.5 ± 1.6
ALP (U/L)	♂	186 ± 17.4	146.5 ± 13	141.7 ± 13.9	157.7 ± 10.4
	♀	211.3 ± 12.0	192.4 ± 7.2	183.6 ± 8.3	212.2 ± 11.6
GLU (mmol/L)	♂	6.6 ± 0.3	5.4 ± 0.4	6.5 ± 0.6	7.7 ± 0.4
	♀	4.99 ± 0.3	4.88 ± 0.2	5.81 ± 0.4	7.59 ± 0.4 ***
VLDL	♂	0.81 ± 0.05	0.84 ± 0.07	0.61 ± 0.08	0.49 ± 0.05 ***
	♀	0.68 ± 0.06	0.56 ± 0.03	0.54 ± 0.05	0.50 ± 0.05
TG (mmol/L)	♂	1.79 ± 0.1	1.84 ± 0.1	1.35 ± 0.1 *	1.09 ± 0.1 ***
	♀	1.51 ± 0.1	1.24 ± 0.08	1.19 ± 0.1	1.19 ± 0.1

All data are presented as the mean ± SEM (*n* = 10). Statistical significance was analyzed by using the Student’s *t*-test. Compared with the control, * and *** represent 0.01 < *p* < 0.05 and *p* < 0.001, respectively. Alanine aminotransferase (ALT); aspartate aminotransferase (AST); albumin (ALB); globulin (GLO); total protein (TP); alkaline phosphatase (ALP); blood glucose (GLU); very low-density lipoprotein (VLDL); triglycerides (TG).

## Data Availability

Not applicable.

## Supplementary Materials

The following supporting information can be downloaded at https://www.mdpi.com/article/10.3390/toxics10060286/s1, Table S1: List of primers used for qPCR. Table S2. Kidneys and liver somatic indexes of HTE in the acute toxicity study. Table S3. The serum biochemical parameters of HTE in the acute toxicity test.
