# Supplementary material for: Acute and Subacute Safety Evaluation of Black Tea Extract (Herbt Tea Essences) in Mice
Source: Toxics. 2022 May 27;10(6):286. doi: 10.3390/toxics10060286 (PMC9228953; doi:10.3390/toxics10060286)
Supplement: Supplementary file 1 [file toxics-10-00286-s001.zip › toxics-1715271-supplementary.pdf]

toxics

MDPI

Supplementary Materials: Acute and subacute safety evaluation of black tea extract (Herbt Tea Essences) in mice

Table S1. List of primers used for qPCR.

Target gene	Primer sequence (5'-3')	Genbank number
M-*Gapdh*-F	TGTGTCCGTCGTGGATCTGA	BC023196.2
M-*Gapdh*-R	TTGCTGTTGAAGTCGCAGGAG	BC023196.2
M-*Il1b*-F	TGACCTGGGCTGTCCTGATG	NM_008361.4
M-*Il1b*-R	GGTGCTCATGTCCTCATCCTG	NM_008361.4
M-*Tnfa*-F	CCCCAAAGGGATGAGAAGTTC	AY423855.1
M-*Tnfa*-R	GGCTTGTCACTCGAATTTTGAGA	AY423855.1
M-*Il6*-F	CTGCAAGAGACTTCCATCCAG	J03783.1
M-*Il6*-R	AGTGGTATAGACAGGTCTGTTGG	J03783.1

Table S2. Kidneys and liver somatic index of HTE in the acute toxicity study.

Organ somatic index (%)	Sex	Control	17.7 g/kg	20.2g/kg
Kidneys	♂	1.78±0.14	1.10±0.06	1.49±0.10
	♀	1.18±0.27	1.43±0.09	1.15±0.02
Liver	♂	5.56±0.18	4.41±0.04	4.63±0.04
	♀	4.61±0.05	4.43±0.02	4.46±0.03

All data are presented as the mean ± *SEM* (n =3). Statistical significance was analyzed by using the student's *t*-test.

Table S3. The serum biochemical parameters of HTE in the acute toxicity test.

Parameter	Sex	Control	17.7 g/kg	20.2 g/kg
BUN(mmol/L)	♂	8.9±3.2	5.2±1.37	7.01±2
	♀	6.4±0.26	7.6±0.83	6.8±1.73
CREA(mmol/L)	♂	16.5±0.9	20.5±0.33	16±0.88
	♀	17.3±0.66	18.9±2.1	19±0.66
BUN/CREA	♂	0.58±0.88	0.30±0.04	0.43±0.08
	♀	0.37±0.03	0.43±0.15	0.37±0.15
UA(μmol/L)	♂	145±17.3	125±2.84	125±43.84
	♀	80.3±10.3	110±26.3	119±19.1
ALT(U/L)	♂	55.3±8.85	37.4±8.27	36.1±7.3
	♀	30.3±2.79	35.3±10.3	37.1±3.04
AST(U/L)	♂	228±34.2	117±14.9	125±8.9
	♀	106±20.3	126±7.17	106±7.6
AST/ALT	♂	4.1±0.47	3.6±0.4	3.67±0.74
	♀	3.5±0.59	3.4±0.58	2.9±0.4
ALB (g/L)	♂	38±1.94	48±1.44	45.7±2.48
	♀	46±0.96	43±0.61	44.9±1.41
GLO (g/L)	♂	16.4±1.25	13.7±2.6	13. 6±0.75
	♀	12.6±0.75	13.8±0.6	14.3±0.72
ALB/GLO	♂	3.69±0.54	3.6±0.81	3.35±0.37
	♀	2.34±0.36	3.11±0.17	3.15±0.25
TG (mmol/L)	♂	54.4±2.2	61.9±0.4	59.4±2.4
	♀	58.6±0.52	57±0.5	59.1±1.3

**ALP (U/L)**	♂	121±28.7	225±41.7	158±49.0
	♀	306±37.8	218±22.5	221±92.6

All data are presented as the mean ± *SEM* (n = 6). Statistical significance was analyzed by using the student's *t*-test.
